# Perinatal Exposure to HIV Infection: The Experience of Craiova Regional Centre, Romania

**DOI:** 10.3390/healthcare10020308

**Published:** 2022-02-06

**Authors:** Eugenia-Andreea Marcu, Sorin-Nicolae Dinescu, Vlad Pădureanu, Florentina Dumitrescu, Radu Diaconu

**Affiliations:** 1 Doctoral School, University of Medicine and Pharmacy of Craiova, 200349 Craiova, Romania; busuandreea01@gmail.com; 2 Department of Epidemiology, University of Medicine and Pharmacy of Craiova, 200349 Craiova, Romania; dinescus73@yahoo.com; 3 Department of Internal Medicine, University of Medicine and Pharmacy of Craiova, 200349 Craiova, Romania; 4 Department of Infectious Diseases, University of Medicine and Pharmacy of Craiova, 200349 Craiova, Romania; 5 Department of Pediatrics, University of Medicine and Pharmacy of Craiova, 200349 Craiova, Romania; raduteodiaconu@yahoo.com

**Keywords:** HIV, perinatal, preterm birth, low birth weight

## Abstract

Background and objectives: HIV infection in pregnant women can be responsible for a number of consequences during pregnancy, such as: maternal anaemia, miscarriage, low birth weight, and preterm birth. The objectives of this study were to determine the maternal–foetal transmission rate of HIV among pregnant women living with HIV from Craiova Regional Centre in order to assess the risk factors for mother-to-child transmission of HIV and to identify the characteristics of newborns perinatally exposed to HIV. Materials and methods: A retrospective study was conducted between 1 January 2011 and 31 December 2020, including children born to HIV-positive mothers. Results: The studied group included 138 newborns and was divided into two subgroups: group A, which included 10 HIV-infected infants; and group B, which included 128 uninfected infants. The mother-to-child transmission rate was 3.5% for women to whom all prophylaxis standards were applied. We found a statistically significant correlation between the level of maternal HIV viremia and perinatal HIV transmission (*p* = 0.01). Preterm birth and low birth weight were associated with perinatal transmission of the infection. Conclusions: Perinatal transmission of HIV infection during our study was associated with inconsistent application of screening for HIV infection among pregnant women, lack of antiretroviral therapy, poor adherence to treatment, and detectable HIV viral load during pregnancy.

## 1. Introduction

According to the World Health Organization (WHO), there were 37.7 million people living with human immunodeficiency virus (HIV) globally on 31 December 2020. By 2020, the estimated number of pregnant women living with HIV across the globe was 1.3 million, and 85% of these women were receiving antiretroviral therapy (ART) to prevent perinatal HIV transmission [1].

In Romania, in June 2021, in conformity with the data of the National Commission for the Fight against AIDS, of the 17,012 people living with HIV, 6589 were women. In 2020, of the 216 infants born to HIV-positive mothers, 213 were given antiretroviral prophylaxis, and six of them acquired the infection [2].

Mother-to-child transmission of HIV can be prevented by diagnosis and treatment of infection in women and adolescents of childbearing age, prenatal screening, proper use of ART in pregnant women to reduce maternal viremia, post-exposure prophylaxis of the newborn, appropriate obstetric attitude, and infant formula feeding [3,4].

HIV infection in pregnant women can be responsible for a number of consequences during pregnancy, such as maternal anaemia, miscarriage, low birth weight, and preterm birth [5].

The association between maternal HIV infection, preterm birth, and low birth weight of infants is a major public health problem [6]. Low birth weight and preterm birth are associated with neonatal mortality and complications.

Among children born to women living with HIV, there is a higher incidence of low birth weight, as well as neonatal and infant mortality, compared to those born to HIV-negative mothers [7].

Compared to the general population, pregnant women living with HIV have a shorter gestation period due to recommendations for prophylactic caesarean section (usually scheduled for week 38), as well as the possibility of preterm birth, facilitated by combined ART, especially with protease inhibitors [8,9,10]. Due to widespread access to ART during pregnancy, preterm birth occurs in about a quarter of pregnant women living with HIV, a proportion similar to that of low-birth-weight infants [11]. Despite the possibility of an increased risk of preterm birth in pregnant women receiving ART, the benefits of this treatment for both the mother and the prevention of perinatal HIV transmission justify the initiation and continuation of treatment during pregnancy [12].

Low birth weight and gestational age less than 34 weeks or more than 38 weeks have been associated with an increased risk of perinatal HIV transmission. The association between prematurity and transmission may be the consequence of HIV infection in utero. Mothers with advanced disease (AIDS) are more likely to give birth to a premature baby [13].

The benefits of ART during pregnancy for reducing the risk of vertical transmission of HIV infection and for the favourable evolution of maternal infection are indisputable. However, studies have shown that pregnant women living with HIV still have significantly higher rates of perinatal complications compared to seronegative pregnant women.

In Romania, according to the Romanian Society of Obstetrics and Gynaecology and the National Commission for the Fight against AIDS, the strategies to prevent mother-to-child transmission of HIV include prenatal screening for all pregnant women, maternal ART during pregnancy and labour, scheduled caesarean section before labour in women who have a level of VL-HIV greater than 400 copies/mL at 36 weeks of gestation, administration of antiretroviral drugs to all newborns who were perinatally exposed to HIV, preferably within 6 h of delivery (Zydovudine syrup + Lamivudine syrup for 6 weeks), and artificial feeding of the newborn [13,14].

The objectives of this study are to determine the maternal–foetal transmission rate of HIV among pregnant women living with HIV registered in Craiova Regional Centre (CRC) in order to assess the risk factors for mother-to-child transmission of HIV and to identify the characteristics of newborns perinatally exposed to HIV.

## 2. Materials and Methods

We performed a retrospective study between 1 January 2011 and 31 December 2020, which included pregnant women diagnosed with HIV infection before pregnancy and intrapartum recorded in the CRC, as well as their newborns.

The inclusion criteria of the participants in our study included infants born to women living with HIV, periodically tested after birth (until 18 months), in our centre. The exclusion criteria of the participants included infants born to women living with HIV in other centres who were lost to follow-up (two infants). 

In our study, among the newborns, we evaluated clinical data (gestational age; anthropometric parameters, such as birth weight, length, and head circumference; and APGAR score), biological data (determination of VL-HIV by polymerase chain reaction—PCR at 6 weeks, 3 months, 6 months, and 12 months; anti-HIV1+2 ELISA test and Western blot HIV after 18 months), data on the prophylactic administration of ART, and the infant-feeding method. Uninfected infants were those with repeated negative virologic tests in the first 6 months or two negative HIV antibody tests after 6 months.

For the mothers in our study group, we analysed epidemiological, demographic, and clinical data, data from laboratory tests (presence of anaemia, serology for co-infections with hepatitis viruses, determination of CD4+ lymphocyte count by flow cytometry, VL-HIV determination), data on the obstetric examination, data on the ART compliance level, data on the duration of treatment, and data on the level of education and socioeconomic status of the mothers. 

The level of compliance with ART was assessed through a compliance questionnaire, which was filled in from time to time by all patients living with HIV from CRC. The questionnaire included questions about the patient’s behaviour towards the treatment plan and the recommendations of the specialist doctor (frequency of administration, number of missed doses per month, which administration was the most difficult, the extent to which correct and uninterrupted administration of ART helped to maintain the patient’s health).

The level of compliance with ART was estimated according to the following scores, by self-reporting [15]:

- 20–22 points = optimal compliance (≥95%)
- 18–19 points = 80% < compliance < 95%
- <18 points = non-compliance (<80%)

The group we studied included 138 newborns from 117 HIV-positive mothers—2 patients had 3 births, and 17 patients had 2 births in the study period. We divided the group of newborns into two other groups: group A, which included 10 HIV-infected infants; and group B, which included 128 seroreverter (uninfected) infants. 

We collected data using medical records of women living with HIV and their newborns, registered within CRC, as well as the electronic database. We used the Analysis ToolPak in Excel for analytical processing and the Fisher test for statistical connections with a significant level if the *p*-value was less than 0.05. For the correlation analysis, we used the Pearson correlation coefficient. To identify the risk factors associated with mother-to-child transmission of HIV, we performed linear regressions. We integrated the results into charts and tables.

All the participants in the study signed an informed consent upon admission for processing of medical data. No other consent was signed by the patients due to the retrospective nature of the study.

The study was conducted according to the guidelines of the Declaration of Helsinki and approved by Ethics Committee of “Victor Babeş” Clinical Hospital of Infectious Diseases and Pneumophthisiology, Craiova, Romania (No. 5004/19 April 2021).

## 3. Results

**Figure 1:**
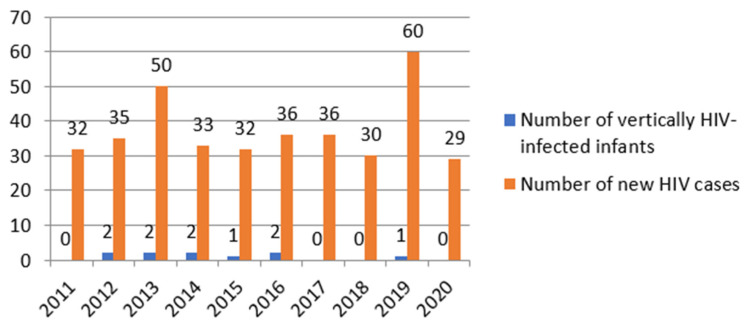
Incidence of mother-to-child transmission of HIV in CRC.

In analysing the status of newborns perinatally exposed to HIV, we detected a mother-to-child transmission rate of 7.2% in the general group. For pregnant women to whom all prophylaxis measures were applied, the mother-to-child transmission rate was 3.5%. The incidence of mother-to-child transmission of HIV was 2,7% during the studied period, with the highest incidence recorded in 2014 (6%) (Figure 1). 

Characteristics of the newborns are presented in Table 1.

The mean birth weight was lower among HIV-positive infants (2520 ± 739.4 g) compared to uninfected infants (2804.4 ± 570.5 g). 

Preterm birth and low birth weight (<2500 g) were associated with mother-to-child transmission of HIV infection. Gestational age <37 weeks was associated with a high risk of low birth weight (*p* = 0.0001).

In the group of HIV-infected children, six had VL-HIV greater than 10,000 copies/mL.

All uninfected infants were administered ART for 6 weeks to reduce the risk of HIV transmission, most of them (122 infants, 95%) receiving a combination of Zidovudine and Lamivudine. The newborns received infant formula. In the group of HIV-infected infants, one newborn did not receive antiretroviral prophylaxis, and two newborns were exclusively breastfed.

The basic characteristics of pregnant women in the studied group are presented in Table 2.

In our study, 83 pregnant women living with HIV (60.1%) were diagnosed with anaemia during pregnancy—mild form in 82% of patients, with an average haemoglobin level of 11.68 ± 1.35 g/dl. We found the presence of coinfections during pregnancy in 24 patients, the most common being chronic hepatitis B (20 cases, 17%). Newborns received immunoprophylaxis and were vaccinated against hepatitis B. None of the babies was vertically infected with hepatitis viruses.

A total of 112 patients (81.1%) used ART for the prevention of perinatal HIV transmission, the most administered therapeutic regimen being Lamivudine/Zidovudine in combination with Lopinavir/Ritonavir. A total of 104 patients received prophylactic ART during the entirety of their pregnancy, and eight patients used ART only in the third trimester. 

Of the 26 women who did not receive ART during pregnancy, six patients (5.1%) gave birth to HIV-positive children. One patient refused the treatment due to poor health literacy, and the other five were diagnosed at birth.

**Figure 2:**
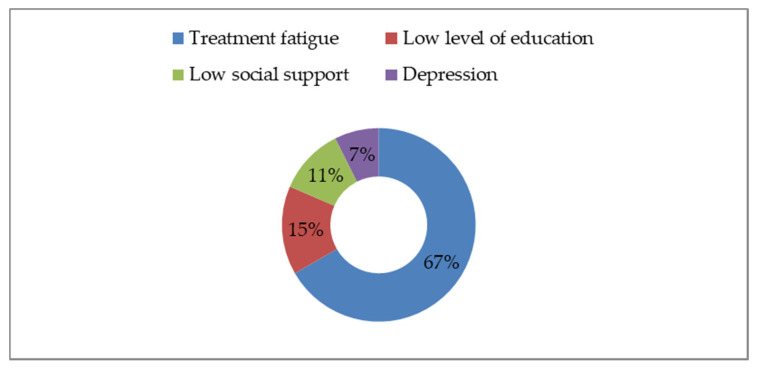
Factors influencing adherence with ART.

Out of the total of 112 women who received ART during pregnancy, we found that 27 (24.1%) were noncompliant with ART (compliance < 80%), and four gave birth to HIV-positive children. Most of those women who were noncompliant with ART come from the Romanian paediatric cohort, which included patients parenterally infected with HIV during early childhood. They had undergone many therapeutic regimens over time, and this led to treatment fatigue and pill burden, the main causes of poor adherence with ART among these patients (Figure 2).

Non-compliance was associated with mother-to-child transmission of HIV, with statistically significant differences between the two groups of newborns (*p* = 0.04).

Regarding the type of birth, 30 patients (21.7%) delivered vaginally, and 108 patients (78.3%) delivered by caesarean section. The birth method was not associated with a high risk of perinatal HIV transmission (*p* = 0.7). Premature rupture of membranes occurred in 31 patients and did not correlate with the risk of mother-to-child transmission of HIV (*p* = 0.08).

Immunological evaluation of pregnant women living with HIV revealed a medium level of immunosuppression, with a median value of 471 cells/mm^3^ [1: 1715] of CD4+ lymphocyte number and 51 women with CD4+ > 500 cells/mm^3^. Pregnant women who gave birth to HIV-positive children had a moderate degree of immunosuppression, with a mean CD4+ lymphocyte count of 315.9 ± 285.3 cells/mm3, with no statistically significant differences between the two groups. The level of immunosuppression was not correlated with the risk of perinatal HIV transmission (*p* = 0.1).

A proportion of 48% of pregnant women had undetectable VL-HIV at delivery. The level of HIV viremia of mothers who transmitted infection was higher than those who delivered uninfected children, with six of the patients having VL-HIV greater than 10,000 copies/mL. We found a statistically significant correlation between the level of VL-HIV and mother-to-child HIV transmission (*p* = 0.01).

**Figure 3:**
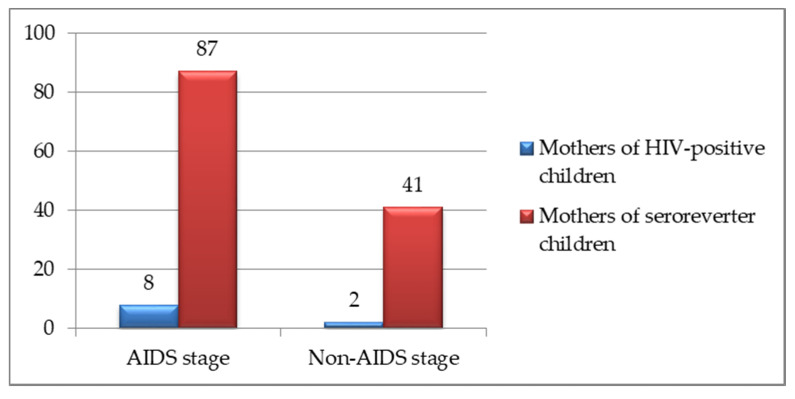
Clinical and immunological classification for HIV infection during pregnancy.

The clinical and immunological classification for HIV infection during pregnancy is presented in Figure 3 (CDC classification system) [16]. The stage of maternal disease was not associated with the risk of perinatal HIV transmission (*p* = 0.8), but it was correlated with preterm birth (*p* = 0.03).

## 4. Discussion

Pregnancy in women living with HIV remains a challenge that requires a multidisciplinary team in order to ensure adequate care. Careful monitoring of the effects of HIV infection on both maternal and newborn health is needed to evaluate and adapt implemented preventive and therapeutic strategies.

A large cohort study was performed in Israel and included women living with HIV who gave birth between January 1988 and December 2011. During the study period, 796 children were born, of whom 25 (3%) acquired the infection. Of all 80 infants who were born before the introduction of ART, 13 (16.3%) were vertically infected, while of the 716 infants who were born after 1997, 12 (1.7%) acquired the infection. Vaginal delivery and lack of ART during pregnancy were associated with mother-to-child transmission of HIV infection [17].

In our study, we identified a rate of vertical HIV transmission of 3.5% among pregnant women living with HIV to whom complete prophylaxis measures were instituted. Maternal–foetal transmission of the infection was associated with low adherence to ART and a detectable level of maternal VL-HIV during pregnancy.

Another study that was conducted in our centre reported a higher vertical transmission rate (9.3%) between 2007 and 2011 due to incomplete prophylaxis measures [18]. 

A study performed in Great Britain between 2008 and 2012 included 61 pregnant women living with HIV, with 19 patients diagnosed with HIV infection during pregnancy. A total of 47% of patients were not on ART, and all patients but one commenced treatment either for prevention of mother-to-child transmission or because they had a CD4 lymphocyte count < 350 cells/mm3. Eight patients (13%) had VL-HIV > 50 copies/mL. A total of 45 patients (74%) gave birth by caesarean section. One newborn was diagnosed with HIV infection. There were six reported preterm births (10%), nine cases of low birth weight (15%), and one stillbirth [19].

Prenatal screening is of great importance, as the diagnosis of HIV infection in pregnant women is essential for prevention of vertical HIV transmission. The diagnosis of HIV infection is correlated with the recommendation of screening for all pregnant women. Repeat HIV testing in the third trimester is recommended for all HIV-negative pregnant women who are known to be at risk of acquiring HIV infection (drug users, women with multiple partners during pregnancy, or HIV-positive partners) [20,21,22,23].

In our study, a significant portion of women living with HIV who transmitted the infection to their children were diagnosed at labour or delivery due to the absence of prenatal screening. Late diagnosis led to a lack of ART, which is one of the main determinants of perinatal HIV transmission.

The infant-feeding method plays a key role in perinatal HIV transmission. According to the WHO, transmission of HIV occurs as a result of breastfeeding in 30–60% of children globally. In high-income countries, it is recommended that mothers living with HIV feed their newborns exclusively with formula milk. In low-income countries, HIV-positive mothers are still advised to breastfeed. The risk of HIV transmission is zero if women living with HIV use infant formula. The absence of ART during breastfeeding and higher VL-HIV increase the risk of mother-to-child transmission of HIV [24]. In Romania, women living with HIV are strongly advised to feed their children with formula milk [14]. 

A screening program for pregnant women has been implemented in the Netherlands since the 1950s. In 2004, universal HIV screening was implemented. A study was performed in order to evaluate the effectiveness of prenatal screening between 2006 and 2008. The estimated annual prevalence of HIV infection among pregnant women was 0.05%. Prior to the introduction of screening, between 5 and 10 newborns were vertically HIV-infected annually. After the introduction of screening in 2004, only four children were born with HIV (an average of one child per year) [25].

Another study performed in Rio Grande, Brazil between 2003 and 2007 included 262 pregnant women living with HIV, with a mean age of 27 years. The rate of mother-to-child transmission of HIV was 3.8%. In 61.5% of cases, children were born by caesarean section. The average weight of the newborns was 2894 g, and the average of APGAR score at five minutes was nine. Regarding the HIV status of the patients, the mean of CD4 lymphocytes was 574 cells/mm3, and the average of viral load at 34 weeks of gestation was 10,603 copies/mL. The mean duration of use of ART was 16 weeks. HIV viremia ≥ 1000 copies/mL and non-use of ART were associated with an increased risk of perinatal HIV transmission. Birth weight ≤ 2500 g, APGAR score ≤ 7, gestational age ≤ 37 weeks, and mother-to-child transmission of HIV were related to inadequate prenatal care [26].

In Romania, the incidence of mother-to-child transmission of HIV was 1,9%, between 2011 and 2020, similar to the incidence of mother-to-child transmission of HIV in our centre. The highest incidence was recorded in 2011 (3%) [27]. 

HIV infection in women who have not received ART is associated with preterm birth and low birth weight. In the study we performed, the proportion of premature infants (gestational age less than 37 weeks) and those with low birth weight was higher among those vertically infected with HIV.

The limitations of our study are the small sample size and the lack of an effective strategy in care and monitoring of pregnant women living with HIV due to poor communication between the obstetrician and the infectious disease doctor. Additionally, there were no data available regarding the mother-to-child transmission rate of HIV in the whole country to make a comparison with the mother-to-child transmission rate of HIV in CRC. 

The implications of our research in current clinical practice consist in developing an effective care strategy and informing all women who want to have a child about the need of HIV testing for primary care. Protocols for pregnant women with unknown HIV status (intrapartum ART, caesarean delivery) must be implemented in departments of obstetrics and gynaecology.

## 5. Conclusions

The incidence of mother-to-child transmission of HIV in CRC was similar to the incidence of mother-to-child transmission of HIV in the whole country.

Perinatal transmission of HIV infection during our study was associated with inconsistent application of screening for HIV infection among pregnant women (women who were diagnosed at delivery), lack of ART, poor adherence to treatment, and detectable HIV viral load during pregnancy.

## Figures and Tables

**Table 1:** Characteristics of the newborns from the studied groups.

Newborn Characteristics		Group A	Group B	Total	*p*
Gestational age	Preterm (<37 weeks)	7	26	33	0.02
	Term (>37 weeks)	3	102	105	
Birth weight	<1500 g	1	2	3	0.04
	1500–2499 g	6	25	31	
	2500 g–3500 g	2	89	91	
	>3500 g	1	12	13	
Length at birth	<10th percentile	5	82	87	0.7
	>10th percentile	5	46	51	
Head circumference	<10th percentile	6	54	60	0.5
	>10th percentile	4	74	78	
APGAR score	<8 points	1	9	10	0.5
	>8 points	9	119	128	

**Table 2:** Basic characteristics of pregnant women living with HIV.

Basic Characteristics		Number of Patients (%)
Mother’s age at first birth	14–19 years old	6 (5.1%)
	20–24 years old	40 (34.2%)
	25–33 years old	67 (57.3%)
	≥34 years old	4 (3.4%)
Place of residence	Urban	24 (20.5%)
	Rural	93 (79.5%)
Education	No studies	4 (3.4%)
	Primary	21 (18%)
	Secondary	44 (37.6%)
	High School	38 (32.5%)
	Higher education	10 (8.5%)
Marital status	Single	36 (30.8%)
	Stable relationship	25 (21.4%)
	Married	56 (47.8%)
Socioeconomic status	Good	81 (69.2%)
	Precarious	36 (30.8%)

## Data Availability

Not applicable.
